# Size- and Oxidation-Dependent Toxicity of Graphene Oxide Nanomaterials in Embryonic Zebrafish

**DOI:** 10.3390/nano12071050

**Published:** 2022-03-23

**Authors:** Ryan M. Lopez, Joshua R. White, Lisa Truong, Robyn L. Tanguay

**Affiliations:** Sinnhuber Aquatic Research Laboratory, Department of Environmental and Molecular Toxicology, Oregon State University, Corvallis, OR 97333, USA; ryan.lopez@oregonstate.edu (R.M.L.); joshuawhitebmb@gmail.com (J.R.W.); lisa.truong@oregonstate.edu (L.T.)

**Keywords:** graphene oxide, zebrafish, developmental toxicity, sodium cholate, protein corona, nanotoxicology, teratogenic, photomotor behavior

## Abstract

Graphene oxides (GOs) are a popular graphene alternative. The goal of this study was to compare the biocompatibility of a diversity of well-characterized GOs. Our previous work advanced developmental zebrafish as a model to interrogate the interactions and biological responses following exposures to engineered nanomaterials (ENMs). Here, we investigated GO 250 nm × 250 nm (sGO), 400 nm × 400 nm (mGO), and 1 μm × 1 μm (lGO), partially reduced GO (prGO) 400 nm × 400 nm, and reduced GO (rGO) 400 nm × 400 nm and 2 μm × 2 μm, which first underwent extensive characterization under the support of the Nanomaterials Health Implications Research (NHIR) Consortium. GOs were stabilized in water (GOs), while prGO and rGOs were dispersed in sodium cholate. Zebrafish were statically exposed to up to 50 μg/mL of each material from 6 h post-fertilization (hpf) until 120 hpf. Toxicity was dependent on GO properties. mGO was the most toxic material; its effects manifested in the yolk syncytial layer (YSL). Additionally, sodium cholate stabilization significantly increased GO toxicity. The observed effects were size- and oxidation-state-dependent, revealing the importance of identifying the structure-specific toxicity of GOs.

## 1. Introduction

Graphene nanomaterials are composed of carbon atoms arranged in a two-dimensional hexagonal lattice, making them useful for applications in advanced electronics due to their high conductivity and large surface area [1]. The high cost of graphene has led to an interest in graphene oxide (GO) as a less-expensive and more versatile alternative. GO is a nanomaterial that can be produced in large quantities through graphite oxidation and subsequent exfoliation [1,2,3]. GO is structurally different from graphene in that it possesses oxygen-containing functional groups (epoxides, hydroxyls, etc.) and often contains structural defects [4,5]. These differences reduce GO’s conductivity and visible light absorbance, but increase the chemical reactivity compared to graphene [6]. Additionally, GO is amphipathic due to its polar functional groups on the periphery of the hydrophobic graphene-like structure, which profoundly affects these materials’ behavior in solution [4].

Due to their ease of modification throughout production, GOs have diverse medical applications. For example, GOs loaded with doxorubicin have been used to deliver the chemotherapeutics directly to cancer cells in vitro [7,8]. GOs are pH sensitive, producing measurable emission spectra changes that are under development for cancer detection [9]. Reduced GO (rGO) and partially reduced GO (prGO) offer additional properties as they possess fewer epoxides and alcohols, but still contain carboxyl functional groups [10]. rGOs can be used as highly sensitive molecular sensors for chemicals of interest, and prGOs can be used as robust filters for unwanted contaminants, such as heavy metals [5,11]. GO’s structural diversity, reactivity, and rapidly expanding applications make it important to understand its potential for unintended impacts on human and environmental health.

Zebrafish (*Danio rerio*) are well established for toxicological applications due to their small size, low husbandry cost, rapid development ex utero, optical transparency early in development, and molecular and genetic similarity to humans [12,13]. As a whole animal model, zebrafish offer systems-level investigation and translatability that is not typically possible with in vitro models [14,15]. Previous GO studies have shown impaired embryonic development of zebrafish due to protein carbonylation and the production of reactive oxygen species (ROS), but our understanding of the importance of GO size- and oxidation-state-dependent toxicity is limited [12,16].

This study evaluated a diverse library of GO materials to determine if size or oxidation state influenced the materials’ biocompatibility using zebrafish high throughput screening. We determined that there was a size- and oxidation-state-dependent toxicity in zebrafish embryos, with GOs dispersed in water being the most toxic. In addition to zebrafish mortality and morphological effects, brightfield imaging showed fine-scale embryonic structural disruptions, in particular at the yolk syncytial layer (YSL), in response to GO exposure. Cell death and reactive oxygen species (ROS) assays were performed to elucidate the GO-specific mechanisms that resulted in zebrafish embryo mortality. In efforts to mitigate the toxicity of GO and understand the translatability from in vitro models, fetal bovine serum (FBS) was added to a GO solution and found to modulate the toxicity of a GO. The structure-specific toxicity identified in this study highlights the need for deeper investigations into the structure-specific bioactivity of GOs.

## 2. Materials and Methods

### 2.1. Zebrafish Husbandry and Embryo Collection

Wild-type zebrafish (5D) housed at Oregon State University’s Sinnhuber Aquatic Research Laboratory (SARL; Corvallis, OR, USA) were used following the protocols approved by the Institutional Animal Care and Use Committee (ACUP 5113). The zebrafish were kept on a 14 h: 10 h, light: dark cycle with 28 °C filtered, recirculating water supplemented with Instant Ocean salts (Spectrum Branks, Blacksburg, VA, USA). Zebrafish were fed with GEMMA micro feed from Skretting Inc. (Fontaine Les Vervins, France) [17].

Zebrafish 5D-lines were spawned in 50-gallon or 100-gallon tanks with approximately 400 zebrafish per tank [17]. The day before embryonic exposure, a spawning funnel was set up. The fertilized eggs were collected the next morning and age-staged into a petri dish with embryo media (EM; 15 mM NaCl, 0.5 mM KCl, 1 mM MgSO_4_, 0.15 mM KH_2_PO_4_, 0.7 mM NaHCO_3_) and stored in an incubator at 28 °C [18,19].

### 2.2. GOs Obtained from Nanomaterials Health Implications Research Consortium

A total of six GOs were received from the Consortium (https://www.niehs.nih.gov/research/supported/exposure/nanohealth; accessed 28 December 2021): GO 250 nm × 250 nm (small GO (sGO)), GO 400 nm × 400 nm (medium GO (mGO)), GO 1 μm × 1 μm (large GO (lGO)), prGO 400 nm × 400 nm, rGO 400 nm × 400 nm, and rGO 2 μm × 2 μm. sGO, mGO, and lGO were dispersed in water (GOs), whereas prGO 400 nm × 400 nm, rGO 400 nm × 400 nm, and rGO 2 μm × 2 μm were dispersed in sodium cholate (Table 1). The synthesis and characterization of these materials were previously described by Parviz and Strano and Bitounis et al., respectively [3,20].

### 2.3. GO Solution Preparation

To minimize GO aggregation in exposure solutions, GO stock solutions were sonicated under temperature-controlled conditions (14 °C) with critical sonication energies ranging from 301.6 J to 1392 J (Table 1) [21,22]. Critical sonication energies describe the amount of energy required to stably suspend GOs in solution [21]. The stability of sonicated GO solutions was monitored via Dynamic Light Scattering (DLS; Malvern Zetasizer Nano ZS ZEN3600, Worcestershire, United Kingdom) and the zeta potential (Malvern Zetasizer Nano ZS ZEN3600). Although DLS is not an ideal technique to use for two-dimensional materials such as GOs, it at least offers a post-sonication baseline from which to monitor any changes over time and a comparison of our data to that of the Consortium (Table S1). Zeta potential values were also compared to that of the Consortium (Table S2: UP). The zeta potentials of GOs dispersed in various media (UP, EM, or UP with sodium cholate) were also evaluated to determine the effects of the dispersion medium on material stabilization (Table S2).

### 2.4. Embryonic GO and Sodium Cholate Exposure

Pronase (product number: 81748; Fluka, St. Louis, MO, USA) was used to remove the chorion of embryos at 4 h post-fertilization (hpf) in an automated dechorionator [23]. Following dechorionation and further incubation until 6 hpf, individual embryos were hand-loaded with flamed wide-bore glass Pasteur pipettes into a 96-well plate containing 100 μL per well of varying concentrations of distinct GO solutions (0 μg/mL, 2.32 μg/mL, 5 μg/mL, 10.7 μg/mL, 23.2, 50 μg/mL), and parafilm was placed between the lid and the plate to limit evaporation. Plates were then placed at 28 °C in an orbital shaker (VWR Model 3500) overnight, shaking at 235 revolutions per minute (rpm) to ensure homogenous exposure to GOs [24]. Embryos were evaluated for mortality at 24 hpf and placed in a 28 °C incubator until 120 hpf when behavioral and morphological assessments were performed. Each GO concentration, including controls, contained 32 animals for robust statistical analyses.

Following the procedure above, the toxicity of sodium cholate was determined using 96-well plates containing 100 μL per well of varying concentrations (0 μg/mL, 0.625 μg/mL, 1.25 μg/mL, 2.5 μg/mL, 5 μg/mL, 10 μg/mL, 25 μg/mL, 50 μg/mL, 125 μg/mL, 250 μg/mL, 300 μg/mL, 400 μg/mL, 500 μg/mL) [23]. Embryos were evaluated for mortality at 24 hpf and placed in a 28 °C incubator until 120 hpf. At 120 hpf, behavioral and morphological assessments were performed. Each concentration of sodium cholate, including the controls, contained 32 animals for robust statistical analyses.

### 2.5. Teratogenicity Endpoints

Incidences of mortality and abnormal morphology were recorded at 24 hpf and 120 hpf for 18 endpoints: eye, snout, jaw, notochord, heart, somite, brain, fin, otic vesicle, yolk sac, trunk, body axis, swim bladder, pectoral fin, caudal fin, pigment [25]. An observation of mortality or a morphological abnormality in any of the 96 wells was recorded as a binary presence/absence in our customized laboratory information management system, known as the zebrafish acquisition and analysis program (ZAAP), which is interfaced with a MySQL database [26]. Data obtained from ZAAP were analyzed using a custom R-script as previously described [26]. Incidences of abnormality or mortality that were significantly above the binomial threshold for significance were considered a “hit” and defined by red dots on the composite plots.

### 2.6. Larval Photomotor Response Behavior Endpoint

The photomotor response behaviors of 120 hpf larvae following GO exposure were assessed using a ZebraBox system that tracked larval movement, and motion analysis was performed by the ZebraLab software (Viewpoint Behavior Technology; Montreal, Québec, Canada). Light/dark (visible/infrared) cycles were 3 min each and repeated 4 times, with only the last light cycle used for data analysis, as it typically has the highest signal:noise ratio. Behavior data analysis was performed using a modified version of a previously described protocol [27].

### 2.7. Window of Susceptibility

Zebrafish embryos exposed to 50 μg/mL of a GO in individual wells of a 96-well plate were screened hourly from 6–30 hpf (24 h) to identify the onset of significant embryo mortality (*n* = 12). Statistical significance was determined using a *t*-test (α = 0.05) that compared UltraPure™ water-exposed (UP; ThermoFisher: catalogue number: 10977015) embryos to GO-exposed embryos.

Epiboly variation experiments were performed after the determination of the window of susceptibility. Embryos were exposed to 50 μg/mL mGO for 1 h at 6 hpf, 8 hpf, and 10 hpf. The 6 hpf corresponded to 50% epiboly, meaning that 50% of the enveloping layer of the embryo covered the yolk sac; 8 hpf and 10 hpf were timepoints that were also chosen to represent 75% and 100% epiboly, respectively [19].

### 2.8. Brightfield Imaging

Images of 6 hpf embryos were taken during the window of susceptibility to mGO using a Keyence BZ-X700 fluorescence microscope to record embryonic structural changes throughout exposure. Imaging in a flat-bottom 96-well plate commenced immediately upon exposure of embryos to 100 μL of 50 μg/mL mGO or UP, 30 min post-exposure, and at the final timepoint of the window of susceptibility for mGO (1 h post-exposure).

### 2.9. Acridine Orange Assay

An acridine orange (AO) (Molecular Probes Inc.; Catalogue number: A1301) assay was used to visualize an apoptotic response in 6 hpf zebrafish embryos following exposure to mGO. First, embryos were exposed to 50 μg/mL of mGO for 30 min, washed 3 times with 100 μL of UP, followed by an addition of 100 μL of AO to a final concentration of 5 μg/mL. The embryos were incubated in AO at room temperature for 1 h, washed 3 times with UP to remove AO residues, and euthanized with tricaine. Embryos were immediately imaged using a rhodamine filter and brightfield imaging in a Keyence BZ-X700 fluorescence microscope.

### 2.10. Dichloro-Dihydro-Fluorescein Diacetate Assay

Dichloro-dihydro-fluorescein diacetate (DCF-DA) (Enzo Clinical Labs; Catalogue number: ALX-610-022-M050) is a non-fluorescent molecule, but upon oxidation, typically through interactions with ROS, a fluorescent intermediate is produced [28]. A DCF-DA assay was employed to confirm ROS production in embryos upon mGO exposure. Dechorionated 6 hpf embryos were exposed to 10 μM DCF-DA dye for 30 min in the dark and then loaded into a round-bottomed 96-well plate containing 100 μL of sonicated mGO solution or UP water. Images were taken every 10 min for 3.5 h using a GFP filter on a Keyence BZ-X700 fluorescence microscope. Mortality was recorded at the end of the experiment, and all surviving embryos were euthanized with tricaine.

### 2.11. Fetal Bovine Serum Combined with GOs

FBS (VWR product number: 89510-194) and mGO co-exposures were performed with 4% FBS while mGO concentrations followed the standard 0 μg/mL, 2.32 μg/mL, 5 μg/mL, 10.7 μg/mL, 23.2 μg/mL, and 50 μg/mL curve (unpublished data). Each well of a 96-well plate contained 100 μL of mGO + 4% FBS solution, as well as a control plate that contained mGO mixed with UP water. All equipment was sanitized with 70% ethanol, and work was performed under a laminar flow hood to maintain a sterile environment. Mortality was monitored at the timepoint determined by the window of susceptibility, and morphology and mortality assessments were performed at 120 hpf. A Welch’s *t*-test (α = 0.05) was used to test for significant mortality and abnormal morphology incidences compared to those of the GO + UP group. Further statistical analysis used a two-way ANOVA to detect the effect that the concentration and medium had on the viability of exposed embryos. Welch’s *t*-test was also employed to test the significance in percent differences from 1 h post-exposure to 24 h post-exposure in the FBS + mGO group against the UP + mGO group.

### 2.12. Ascorbic Acid Mixed with GOs

Initially, the toxicity of ascorbic acid (AA) was determined using 6 hpf embryos; previous studies showed that the maximum tolerated concentration of AA was ≤250 μM (exposures started at 3 hpf), 180–360 μM (exposures started at 4 hpf), or 100–200 μM (exposures started at 0 hpf) [29,30,31]. We determined that the maximum tolerated concentration for dechorionated embryos was 100 μM only for 1 h post-exposure.

Zebrafish embryos (*n* = 24) were co-exposed, from 6–120 hpf, to 107 μM ascorbic acid (Sigma-Aldrich, St. Louis, MO, USA; Product #: 255564-5G) and 10.7 μg/mL mGO in a 96-well plate; 107 μM was chosen instead of 100 μM to reach a 2:1 (AA:mGO) ratio as closely as possible. The same plate also contained embryos (*n* = 24) exposed to mGO, AA, or UP alone. Embryo viability was recorded at 7 hpf, 24 hpf, and 120 hpf. Statistical significance was tested using Welch’s *t*-test (α = 0.05).

## 3. Results

### 3.1. Developmental Toxicity Screening of GOs

#### 3.1.1. Sodium Cholate Maximum Tolerated Concentration Was 400 μg/mL

**Figure 1:**
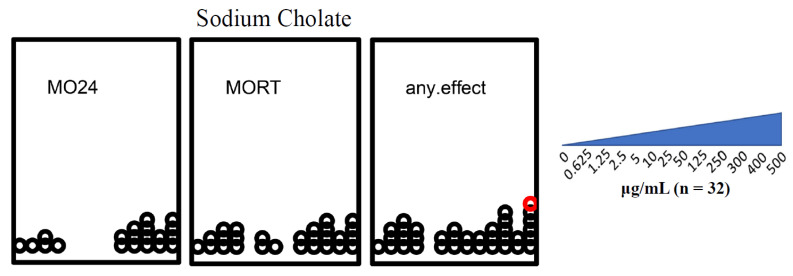
Developmental toxicity of sodium cholate. MO24 = 24 hpf mortality; MORT = 120 hpf mortality; any.effect = physical malformations, including mortality at 120 hpf. Outcomes significantly above the binomial threshold (Student’s *t*-test; α = 0.05) are indicated in red on the dot plot. The sodium cholate concentration gradient increases from left to right (0 μg/mL, 0.625 μg/mL, 1.25 μg/mL, 2.5 μg/mL, 5 μg/mL, 10 μg/mL, 25 μg/mL, 50 μg/mL, 125 μg/mL, 250 μg/mL, 300 μg/mL, 400 μg/mL, 500 μg/mL).

Sodium cholate toxicity was assessed before the testing of cholate-dispersed GOs. Exposures up to 400 μg/mL sodium cholate were not associated with significant incidences of teratogenicity. However, teratogenicity incidences reached the significance threshold in the animals exposed to 500 μg/mL sodium cholate (*p* < 0.05) (Figure 1). All subsequent testing of cholate-dispersed GOs was normalized to 400 μg/mL sodium cholate.

#### 3.1.2. sGO Was Not Teratogenic

**Figure 2:**
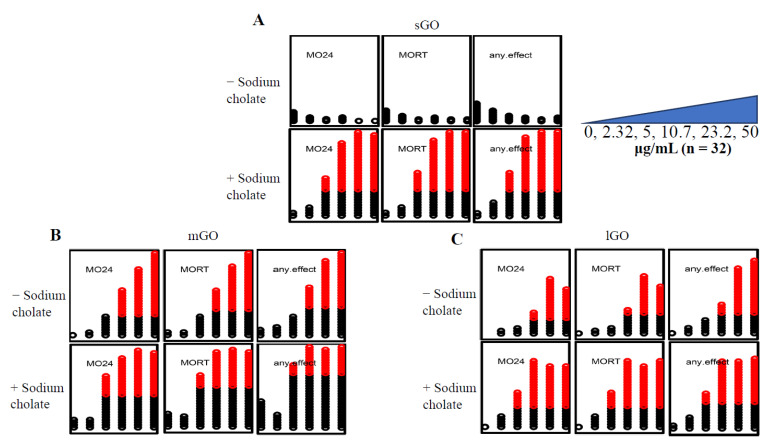
Comparison of GOs with and without sodium cholate. The GO concentration gradient increases from left to right (0 μg/mL, 2.32 μg/mL, 5 μg/mL, 10.7 μg/mL, 23.2 μg/mL, 50 μg/mL), each concentration contained dechorionated embryos (*n* = 32) exposed for 120 h. MO24 = 24 hpf mortality; MORT = 120 hpf mortality; any.effect = physical malformations, including mortality at 120 hpf. Outcomes significantly above the binomial threshold (Student’s *t*-test; α = 0.05) are indicated in red on the dot plot. (**A**) sGO exposures show an increase in mortality upon addition of sodium cholate. (**B**) mGO exposures show an increase in mortality when sodium cholate was added to the solution. (**C**) Addition of sodium cholate to lGO (lGOC) had a wider range of mortality compared to lGO (without sodium cholate).

Exposure of 6 hpf embryos to three GOs (sGO, mGO, or lGO) caused developmental toxicity dependent on the size of the nanomaterials. sGO exposures did not cause significant mortality to dechorionated embryos (*n* = 32) at any of the concentrations investigated (0 μg/mL, 2.32 μg/mL, 5 μg/mL, 10.7 μg/mL, 23.2 μg/mL, 50 μg/mL) (Figure 2A). mGO and lGO exposures caused concentration-dependent mortality at both 24 hpf and 120 hpf, with significant mortality beginning at 10.7 μg/mL in both GO exposures (*p* < 0.05) (Figure 2B,C).

#### 3.1.3. GO Co-Exposure with Sodium Cholate Enhanced Teratogenicity

GO co-exposures with 400 μg/mL of sodium cholate (denoted as -GOC) demonstrated increased incidences of teratogenicity, specifically mortality, compared to GO alone (Figure 2). sGOC co-exposures caused significant mortality (*p* < 0.05), but sGO alone did not (Figure 2A). The lowest effect level (LEL) of both mGO and lGO was reduced by co-exposure to sodium cholate, from 10.7 μg/mL to 5 μg/mL (Figure 2B,C).

#### 3.1.4. Only 2 μm × 2 μm rGO and prGO in Sodium Cholate Were Teratogenic

**Figure 3:**
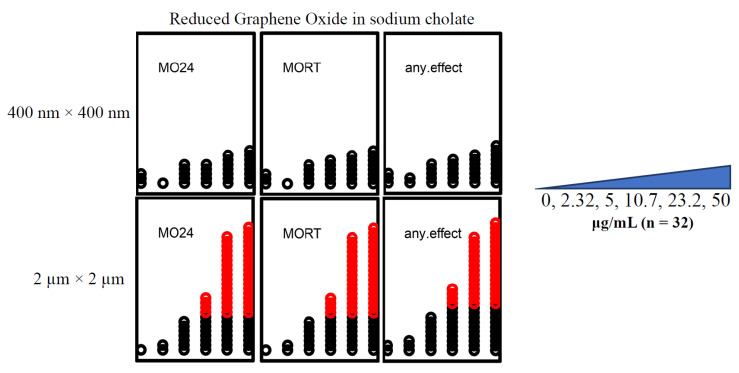
rGO developmental toxicity. rGO exposures of embryos (*n* = 32) revealed that the 400 nm × 400 nm rGO did not cause significant mortality at any of the concentrations investigated (0 μg/mL, 2.32 μg/mL, 5 μg/mL, 10.7 μg/mL, 23.2 μg/mL, 50 μg/mL) (*p* > 0.05). The 2 μm × 2 μm rGO exposures caused mortality and malformations starting at 10.7 μg/mL. MO24 = 24 hpf mortality; MORT = 120 hpf mortality; any.effect = physical malformations, including mortality at 120 hpf. Outcomes significantly above the binomial threshold (Student’s *t*-test; α = 0.05) are indicated in red on the dot plot.

**Figure 4:**
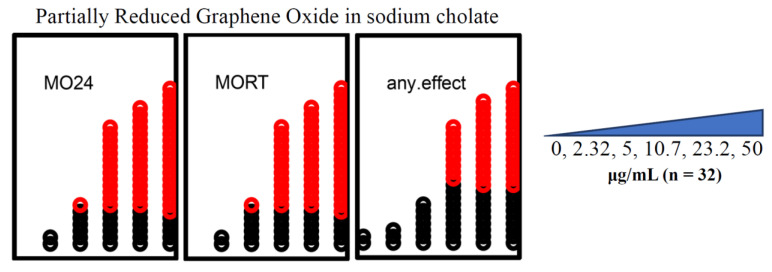
prGO developmental toxicity. Embryonic exposures to 0 μg/mL, 2.32 μg/mL, 5 μg/mL, 10.7 μg/mL, 23.2 μg/mL, and 50 μg/mL prGO (*n* = 32) caused significant mortality at both 24 hpf and 120 hpf (*p* < 0.05). The calculated LEL was 10.7 μg/mL. MO24 = 24 hpf mortality; MORT = 120 hpf mortality; any.effect = physical malformations, including mortality at 120 hpf. Outcomes significantly above the binomial threshold (Student’s *t*-test; α = 0.05) are indicated in red on the dot plot.

Developmental exposures to 2 μm × 2 μm rGO resulted in higher embryonic mortality than did exposures to 400 nm × 400 nm rGO. There was significant mortality associated with exposures to 10.7 μg/mL of the 2 μm × 2 μm rGO (*p* < 0.05), whereas 400 nm × 400 nm rGO exposures did not cause significant mortality at any of the concentrations tested (Figure 3). Exposures to prGO (400 nm × 400 nm) resulted in a LEL estimate of 10.7 μg/mL, driven entirely by mortality (Figure 4).

#### 3.1.5. GO Exposures Impacted Larval Photomotor Behavior

Exposures to GOs in developing zebrafish impacted larval photomotor response (LPR) behavior in a size-dependent manner (Table 2). Larval zebrafish exposed to sGO were hyperactive in the dark phase (normally more active) for all concentrations tested (2.32 μg/mL, 5 μg/mL, 10.7 μg/mL, 23.2 μg/mL, 50 μg/mL) compared to unexposed larvae (Table 2; Figure S2A). mGO exposures exhibited hyperactivity at the 2.32 μg/mL and 5 μg/mL concentrations, while lGO exposures resulted in hypoactive larvae at the same concentrations (Figure S2B,C, respectively).

rGO exposures resulted in concentration-dependent and size-specific behavioral changes (Figure S3). In the 400 nm × 400 nm rGO exposures, abnormal behavior was observed starting at 5 μg/mL (Figure S3A). The 2 μm × 2 μm-rGO-exposed animals displayed abnormal behavior starting at 2.32 μg/mL (Figure S3B). Behavioral assays were limited to the 2.32 μg/mL and 5 μg/mL rGO 2 μm × 2 μm exposures to avoid any mortality identified in previous assessments. No behavioral changes were observed in any of the prGO exposures below the LEL (10.7 μg/mL) compared to unexposed embryos (Figure S4).

### 3.2. Adverse Outcomes

#### 3.2.1. GOs Impacted Development Early in the Exposure Window

**Figure 5:**
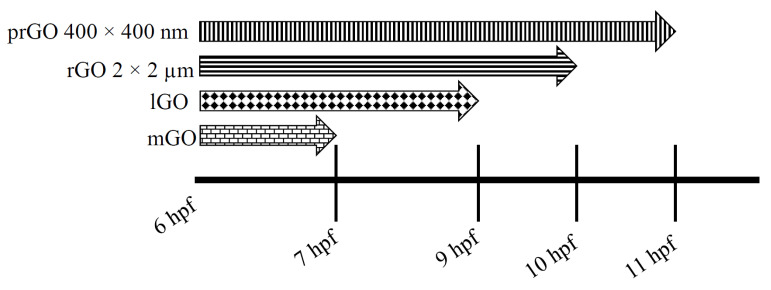
Window of susceptibility starting at 6 hpf. All GOs were tested at 50 μg/mL. mGO (brick pattern) caused the most rapid mortality with significant mortality observed at 7 hpf (*p* = 0.037). lGO (diamond pattern), rGO 2 μm × 2 μm (horizontal stripes), and prGO 400 nm × 400 nm (vertical stripes) caused significant mortality 9 hpf (*p* = 0.027), 10 hpf (*p* = 0.012), and 11 hpf (*p* = 0.012) (respectively). Significant mortality at specified timepoints was determined by Welch’s *t*-tests (α = 0.05).

The onset of significant mortality was GO-specific. Exposure to 50 μg/mL of lGO, mGO, rGO 2 μm × 2 μm, and prGO 400 nm × 400 nm caused significant embryo mortality within 30 hpf (Figure 5). mGO exposures induced the most rapid embryo mortality with significant mortality observed by 7 hpf (*p* = 0.037), but prGOs (dispersed in sodium cholate) of the same size (400 nm × 400 nm) caused mortality by 11 hpf (*p* = 0.012). lGO exposures caused significant mortality by 9 hpf (*p* = 0.027), and rGO 2 μm × 2 μm exposures caused significant mortality by 10 hpf (*p* = 0.012). Therefore, the ranking of the nanomaterials based on their time until the onset of mortality was: mGO, lGO, rGO 2 μm × 2 μm, and prGO 400 nm × 400 nm.

Later-life-stage embryos (8–48 hpf) exposed to 50 μg/mL mGO had a higher survival rate than earlier-life-stage embryos. No significant incidences of mortality occurred in 24 hpf and 48 hpf embryos exposed to 50 μg/mL mGO or lGO for 30 min, 1 h, 2.5 h, and 3 h (*n* = 22; *p* > 0.05). Exposures to 50 μg/mL mGO beginning at 8 hpf and 10 hpf did not cause significant mortality when compared to 6 hpf mGO exposures (*n* = 12 per treatment). Comparing the treatments to their unexposed counterparts of the same age, significant mortality occurred in 6 hpf embryos exposed for 1 h (42% mortality; *p* = 0.017), but not in the 8 hpf and 10 hpf embryos exposed for 1 h (25% mortality; *p* = 0.082 and 0.8% mortality; *p* = 0.34, respectively).

#### 3.2.2. Structural Malformations

**Figure 6:**
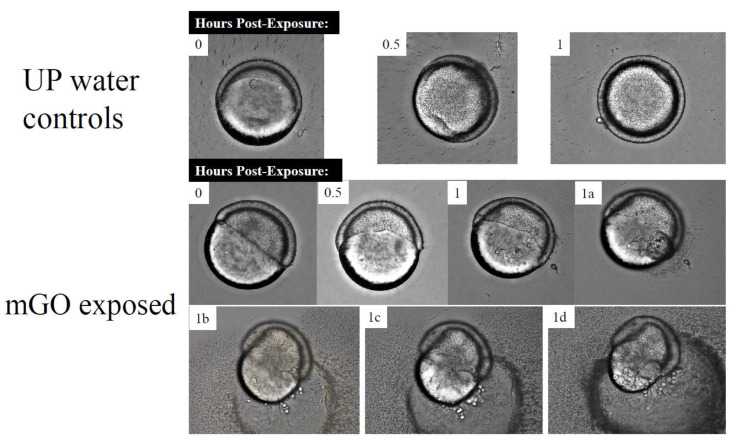
Brightfield imaging of 6 hpf embryos exposed to mGO. The 50 μg/mL-mGO-exposed and UP water control embryos imaged for 1 h after initial exposure at 6 hpf. Images were taken on BZ-X700 Keyence microscope in a flat-bottom 96-well plate. Images 1a, 1b, 1c, and 1d in the mGO-exposed images were taken in sequential order several minutes apart after the 1 h post-exposure timepoint.

Since mGO quickly elicited significant teratogenicity, this nanomaterial was singled out as a worst-case representative of GO developmental hazard. Following exposure to 50 μg/mL mGO for 1 h, membrane disruption of the embryonic yolk sac was observed (Figure 6). Membrane disruption caused the yolk contents to leak out, leading to embryo mortality.

#### 3.2.3. mGO Exposures Did Not Produce ROS

**Figure 7:**
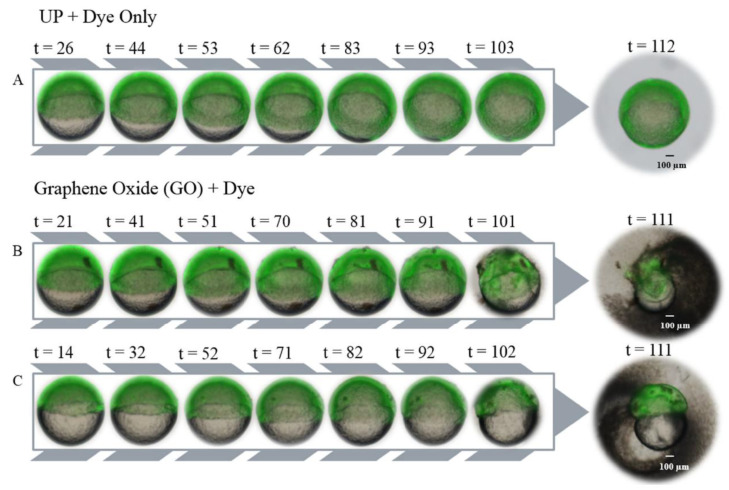
ROS fluorescence imaging of mGO and UP water control embryos. After DCF-DA incubation for 30 min, 6 hpf embryos were exposed to 50 μg/mL mGO or UP water and imaged every 10 min. (**A**) Representative embryo in UP water. The embryo oriented itself so that yolk sac was no longer visible after 83 min post-exposure. (**B**) Embryos exposed to mGO. Observed accumulation of GO particles on the surface of the embryo. Yolk content loss is visible in later timepoints of the images. (**C**) Another embryo exposed to mGO. We can see the accumulation of GO particles on the surface of the embryo. t = minutes post-exposure.

Several studies have shown that ROS production leads to GO toxicity. Here, 6 hpf embryos exposed to mGO did not manifest cellular ROS production different from that of 6 hpf controls embryos in water. DCF-DA, a fluorescent dye commonly used to detect cellular ROS, was used to visualize ROS production following mGO exposure (Figure 7). Embryos in water fluoresced throughout the animal pole, where cellular division is highly active and does not contain yolk, which confirmed that DCF-DA infiltrated the embryos. However, there were no differences in embryo fluorescence between mGO exposure or water controls (Figure 7). Over the course of the imaging, mGO aggregation was observed on the surface of exposed embryos, in particular at the final timepoints investigated (t = 81–102), causing the dark spots in those images (Figure 7B,C).

#### 3.2.4. mGO Did Not Induce Cell Death

Several studies have also identified apoptosis as a cause of GO toxicity. Exposure of 6 hpf embryos to 50 μg/mL mGO for 30 min did not produce a significant apoptotic signal (Figure S5). Similar to the ROS assay, a non-specific fluorescent signal was identified throughout both the mGO-exposed and the UP water control embryos.

### 3.3. Mitigation of mGO Developmental Toxicity

#### 3.3.1. mGO Exposure with Fetal Bovine Serum

**Figure 8:**
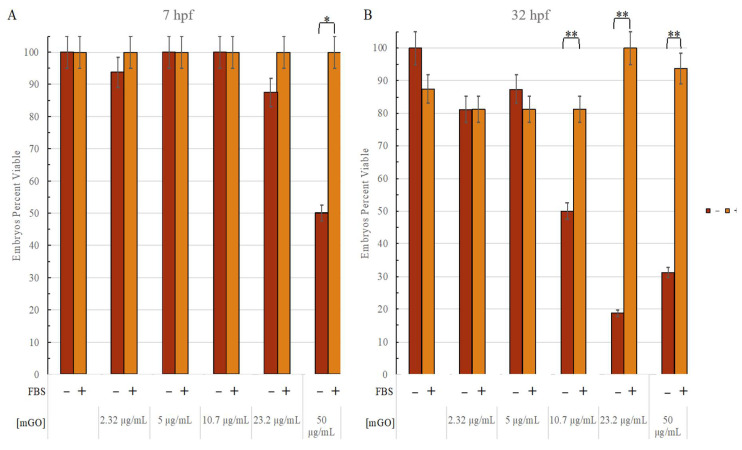
UP + mGO toxicity compared to FBS + mGO toxicity for 26 hpf. Red bars indicate the percent viability for the embryos that were exposed to mGO alone; orange bars indicate the percent viability for mGO + 4% FBS-exposed embryo. Concentrations found below the UP and FBS groups indicate the increasing mGO concentration in the solution (0 μg/mL, 2.32 μg/mL, 5 μg/mL, 10.7 μg/mL, 23.2 μg/mL, 50 μg/mL). Minor lines on the y-axis indicate a 5% change. (**A**) There was a significant difference between the mGO-only group and the FBS + mGO group at the highest concentration (50 μg/mL) 1 h post-exposure (7 hpf). (**B**) The 32 hpf magnifies the trends that were starting to appear at 7 hpf. As observed in the range-finding portion of this study, 10.7 μg/mL was the LEL in the embryos exposed to mGO only. On the other hand, this effect was not reflected in the embryos exposed to the FBS mixture. *: *p* ≤ 0.01. **: *p* ≤ 0.001.

Since most ENM toxicity assessments are performed on cultured mammalian cells that are commonly supplemented with FBS, we sought to compare the zebrafish endpoint results more directly to that of mammalian culture endpoints by adding FBS in the GO exposures. When FBS was added to mGO prior to 6 hpf zebrafish exposures, FBS mitigated mGO-induced mortality in a time-dependent manner (Figure 8). At 7 hpf, the 50 μg/mL mGO + 4%-FBS-exposed 7 hpf embryos were 100% viable compared to 50% viability in exposures to mGO alone. At 30 hpf, mGO + FBS-exposed embryos showed significantly higher survival in 10.7 μg/mL, 23.2 μg/mL, and 50 μg/mL exposures, compared to embryos of the same age that were exposed to mGO alone (81.25% vs. 50%; 100% vs. 18.75%; 93.75% vs. 31.25% respectively). Overall, there was a significant increase in animal viability in the presence of 4% FBS (*p* < 0.002), and FBS-mediated protection against mortality was heightened in higher concentrations (10.7 μg/mL, 23.2 μg/mL, and 50 μg/mL) of mGO at both timepoints investigated (ANOVA: *p* < 0.001). The difference between 7 hpf and 30 hpf in FBS-mediated protection was significant for mGO + FBS exposures at 23.2 μg/mL when compared to mGO + UP exposures under the same conditions (*p* < 0.0001).

#### 3.3.2. Ascorbic Acid Did Not Mitigate and mGO Teratogenicity

Ascorbic acid (AA), a radical scavenger molecule, did not reduce mGO-associated mortality. AA and mGO were combined in a 1.76:1 (final concentrations: 107 μM AA:10.7 μg/mL mGO) mixture prior to 6 hpf embryo exposure. The AA + mGO mixture did not reduce embryonic mortality compared to mGO alone.

## 4. Discussion

Consistent with prior studies, GOs exhibited size- and oxidation-state-dependent toxicity in early-life-stage zebrafish [32,33,34]. However, the queries made here into the potential mechanisms of toxicity were largely negative; exposure of 6 hpf zebrafish embryos to GOs for 1 h did not produce ROS, nor result in an apoptotic response. Since there was a lack of a signal for both of the assays, there may be another mechanism at play, such as lipid peroxidation. In addition to the possibility of another mechanism, the rapid mortality from mGO exposures made it difficult to use ROS or apoptotic assays for our model. The lack of ROS production in this study was unexpected as previous reports have shown that GOs induce ROS-mediated mortality through oxidative stress in bacterial and mammalian cell cultures [32,33]. Another study found that in vitro zebrafish gill tissue produced ROS in response to GO exposure and subsequently altered the gill morphology [35]. Other studies demonstrated that GOs induced cell death through lipid peroxidation and membrane damage in bacterial and human cell cultures [32,34,36,37].

The presence of sodium cholate in GO dispersions significantly increased their teratogenicity. Sodium cholate is an established dispersant and stabilizer for nanoparticles and insoluble organic compounds for both in vivo and in vitro systems [38,39,40]. The increase in GO mortality could be from sodium cholate aiding the delivery of GO into the embryo and amphipathic GO association with the lipid-rich environment of the embryo. The addition of sodium cholate to other micelle systems, such as mPEG-PDLLA, has been observed to improve the delivery of chemotherapeutics to tumors [41].

Our results suggest that GO-induced mortality was driven by damage to the yolk sac. Exposure to mGO that began at 50%, 75%, and 100% epiboly (6 hpf, 8 hpf, and 10 hpf respectively) seemed to be negatively correlated with YSL disruption. Other studies have shown that chemicals and nanomaterials, such as estradiol and GO, accumulate in the yolk sac [42]. Graphene-based materials may enter the yolk through lipid peroxidation and membrane damage [34,36]. As previously described, radical carbons are found throughout the entire molecular structure of GOs, which could cause lipid peroxidation in cells [36]. Additionally, GOs induced mortality through interactions with the membrane surface of naive and antibiotic-resistant bacteria [36]. It is possible that, due to lipid peroxidation of the membrane of the YSL, holes were formed, which leaked yolk. When the yolk began leaking, other GO particles could have quickly associated with the hydrophobic pool that formed and caused further peroxidation and membrane damage around the first tear formed. The YSL is a rapidly developing area, which could trap GOs in a thin (10 μm), quickly developing membrane and result in lipid peroxidation [43].

The GO oxidation level likely played a role in the rapidity of mortality, where fully oxidized GO exposures (mGO, lGO) caused more rapid mortality than the less-oxidized materials (prGO 400 nm × 400 nm and rGO 2 μm × 2 μm). Interestingly, there were size-specific differences in GO toxicity, even in materials such as mGO and lGO that possess the same molecular structures. According to Komeily-Nia et al., the carbon:oxygen (C:O) ratio determines the density of radical carbons in GO structures [44]. The NHIR GO materials we investigated might have possessed a high radical content via the synthetic process chosen by the chemists. The levels of radical content for GOs can be modulated based on the combination of oxidation and reduction methods [44,45]. In support of our observations, prGO, which is more oxidized, should contain more carbon radicals than rGOs of the same size, explaining the difference in mortality we observed. The C:O ratio of mGOs used in our investigations could indicate an abundance of immobilized free radicals that would induce lipid peroxidation of the YSL of zebrafish. Komeily-Nia and colleagues stated that graphite oxide possessed a higher radical density when the C:O ratio was 3 [44]. The sGO, mGO, and lGO used in this study had C:O ratios of 1.633, 1.357, and 1.415, respectively; prGO, rGO 400 nm × 400 nm, and rGO 2 μm × 2 μm contained a C:O ratio of 2.599, 3.545, and 3.484, respectively. The C:O ratios in our study are comparable with what Komeily-Nia et al. observed, but their study primarily focused on graphite oxide and the carbon radical density in response to further oxidation treatments [44]. GOs with C:O ratios that were approximately 1 were associated with significant mortality, but exposure to partially and fully reduced GOs with a C:O ratio of about 3 were associated with higher incidences of mortality. Further investigations on the immobilized free radical density values for GOs are needed to accurately correlate our observations with the free radical density of the GO plane. Previous studies have focused on the C:O ratio and the effect on the carbon radical density in graphite oxide, but more research on how this ratio affects GO’s radical density is needed. Further investigations into the relationship of carbon radical densities in GOs and the occurrence of embryo mortality are also needed to evaluate this relationship.

The interactions between FBS and mGO significantly reduced embryonic mortality. FBS contains a variety of compounds such as amino acids, standard cell culture medium, and vitamins [46]. The mixture of FBS compounds essential for cells in culture likely interacts with GOs to shield the amphipathic nature of the GOs from interacting with the lipids of the cell membrane, blocking their ability to cause lipid peroxidation and other effects. Franqui and colleagues found that when 10% FBS was added to single-layer GOs in DMEM, the FBS proteins formed two coronal layers with the GO [47]. The effect of FBS on the toxicity of mGO would suggest that mammalian cell culture conditions may mask the potential hazard of GOs and other nanomaterials.

## 5. Conclusions

GO toxicity in the developing zebrafish depended on the size and oxidation level. GO 400 nm × 400 nm (mGO) was the most toxic. The mechanism may be lipid peroxidation and membrane damage by immobilized free radicals, especially in the more oxidized GOs [33,43]. This mechanism would seem plausible at the YSL of the zebrafish embryo. Future studies will focus on the potential for lipid peroxidation with more sophisticated detection techniques and markers in zebrafish developmentally exposed to mGO. Additional studies are also needed to evaluate whether developmental behavior effects also manifest in some form in adult zebrafish, e.g., as social or cognitive deficits.

## Figures and Tables

**Table 1:** GO characterization data. Six GOs were provided by the NHIR Consortium. Three were dispersed in water (GO 250 nm × 250 nm, 400 nm × 400 nm, and 1 μm × 1 μm); the remaining three were dispersed in sodium cholate (prGO 400 nm × 400 nm; rGO 400 nm × 400 nm, 2 μm × 2 μm). Stock concentrations were determined using Beer’s law. X-ray photoelectron spectroscopy (XPS) was used to determine the atomic ratio found in the GO structure.

Graphene Oxide Full Name	Short Name	Stock Concentration (μg/mL)	Sodium Cholate Concentration (μg/mL)	Carbon Percent	Oxygen Percent	Critical Sonication Energy (J)
GO 250 nm × 250 nm in water	Small GO (sGO)	500	N/A	62	37.5	1392
GO 400 nm × 400 nm in water	Medium GO (mGO)	310	N/A	57	42	301.6
GO 1 μm × 1 μm in water	Large GO (lGO)	500	N/A	58	41	696
prGO 400 nm × 400 nm in sodium cholate	prGO	500	4000	72	27.7	301.6
rGO 400 nm × 400 nm in sodium cholate	rGO 400 nm × 400 nm	500	2500	78	22	301.6
rGO 2 μm × 2 μm in sodium cholate	rGO 2 μm × 2 μm	400	2500	77.7	22.3	301.6

**Table 2:** Summary of Behavioral outcomes in response to GO exposures. Tabulated summary of the behavior effects that were recorded in graphical form (see Figures S2–S4) obtained when the embryos were 5 dpf. An asterisk (*) indicates that the activity level is dependent on the concentration that the embryos were exposed to at 6 hpf.

GO	Concentration	Behavioral Outcome
sGO	2.32, 5, 10.7, 23.2, 50	Hyperactive
mGO	2.32, 5	Hyperactive
lGO	2.32, 5	Hypoactive
prGO	2.32, 5	No bevavioral changes
rGO 400 nm × 400 nm	2.32	No behavioral changes
	5, 10.7, 23.2, 50	Abnormal *
rGO 2 μm × 2 μm	2.32, 5	Abnormal *

## Data Availability

The data presented in this study are available on request from the corresponding author.

## Supplementary Materials

The following are available online at https://www.mdpi.com/article/10.3390/nano12071050/s1, Figure S1: AFM images of GOs. (A) sGO. (B) mGO. (C) lGO. (D) rGO 400 nm × 400 nm. (E) rGO 2 μm × 2 μm, Figure S2: Average movement of 120 hpf zebrafish exposed to GOs. (A) sGO. (B) mGO. (C). lGO, Figure S3: Average movement of 120 hpf zebrafish exposed to rGOs. (A) rGO 400 nm × 400 nm. (B) rGO 2 μm × 2 μm, Figure S4: Average movement of 120 hpf zebrafish exposed to prGOs, Figure S5: AO in 6 hpf embryos and 24 hpf larvae treated with 50 μg/mL mGO or the control UP water. Top left: Representative UP control embryo incubated at room temperature with AO for 1 h. Widespread signal found in embryo. Top right: Representative embryo developed in UP water. Bottom left: Representative 6 hpf embryo exposed to mGO for 30 min, followed by five washings with UP and AO exposure for 1h. AO accumulation occurred with the lipophilic yolk contents, but there were no localized apoptotic signals detected. Bottom right: Representative embryo exposed to 50 μg/mL mGO for 30 min and washed with UP water five times, Table S1: Zeta potential of sGO, mGO, lGO dispersed in various media (UltraPure (UP), Embryo Media (EM), or UP with sodium cholate (400 μg/mL), Table S2: Average hydrodynamic radius of GOs.
