# Supplementary material for: Size- and Oxidation-Dependent Toxicity of Graphene Oxide Nanomaterials in Embryonic Zebrafish
Source: Nanomaterials (Basel). 2022 Mar 23;12(7):1050. doi: 10.3390/nano12071050 (PMC9000472; doi:10.3390/nano12071050)
Supplement: Supplementary file 1 [file nanomaterials-12-01050-s001.zip › nanomaterials-1595138-supplementary.pdf]

nanomaterials

MDPI

Supplementary Materials

Size- and Oxidation-dependent Toxicity of Graphene Oxide Nanomaterials in Embryonic Zebrafish

Ryan M. Lopez, Joshua R. White, Lisa Truong and Robyn L. Tanguay *

Sinnhuber Aquatic Research Laboratory, Department of Environmental and Molecular Toxicology, Oregon State University, Corvallis, OR 97333, USA; ryan.lopez@oregonstate.edu (R.M.L.); joshuawhitebmb@gmail.com (J.R.W.); lisa.truong@oregonstate.edu (L.T.)

* Correspondence: robyn.tanguay@oregonstate.edu

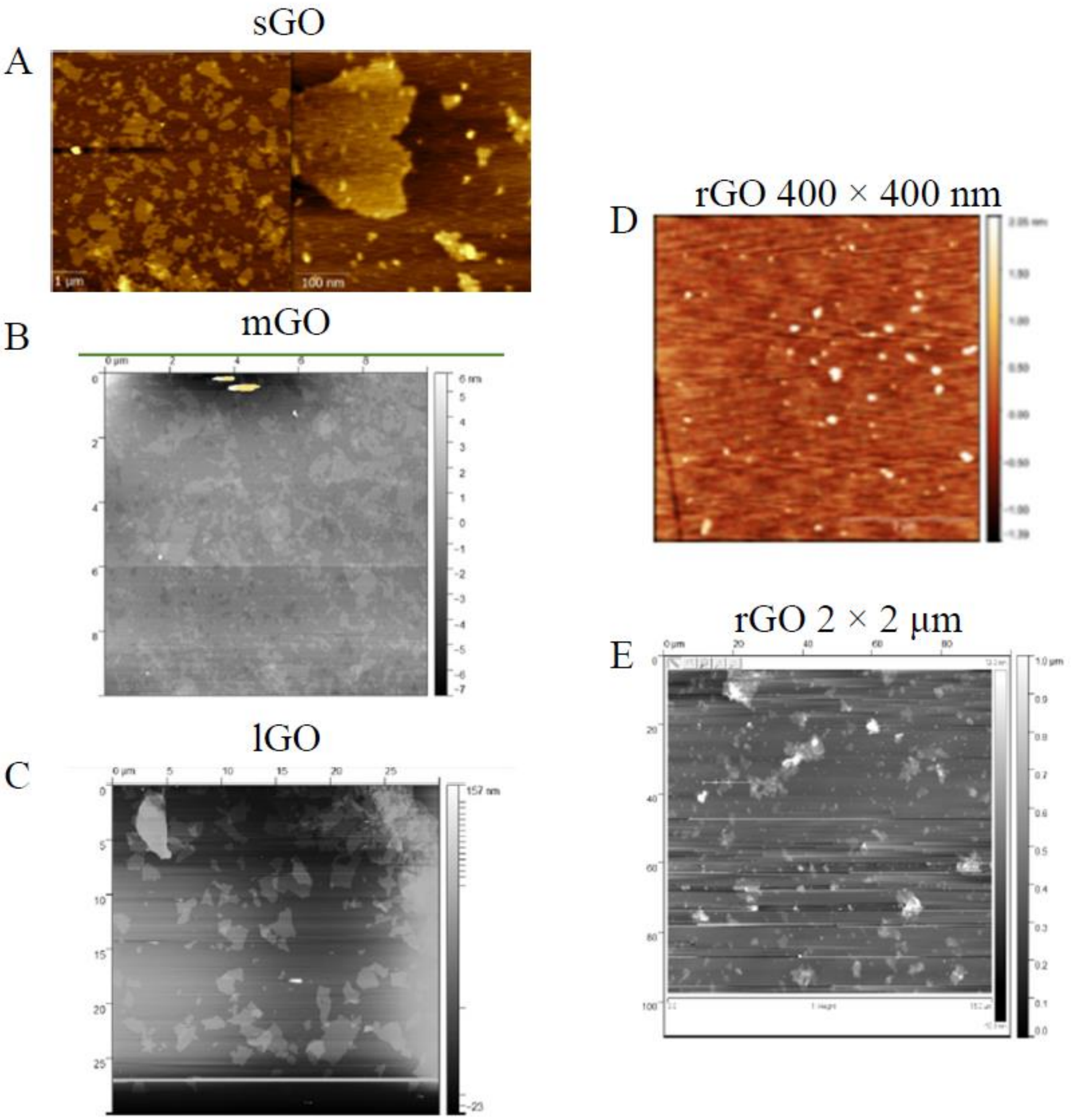

Figure S1. AFM images of GOs. (**A**) sGO, (**B**) mGO, (**C**) lGO, (**D**) rGO 400 × 400 nm, (**E**) rGO 2 × 2 µm.

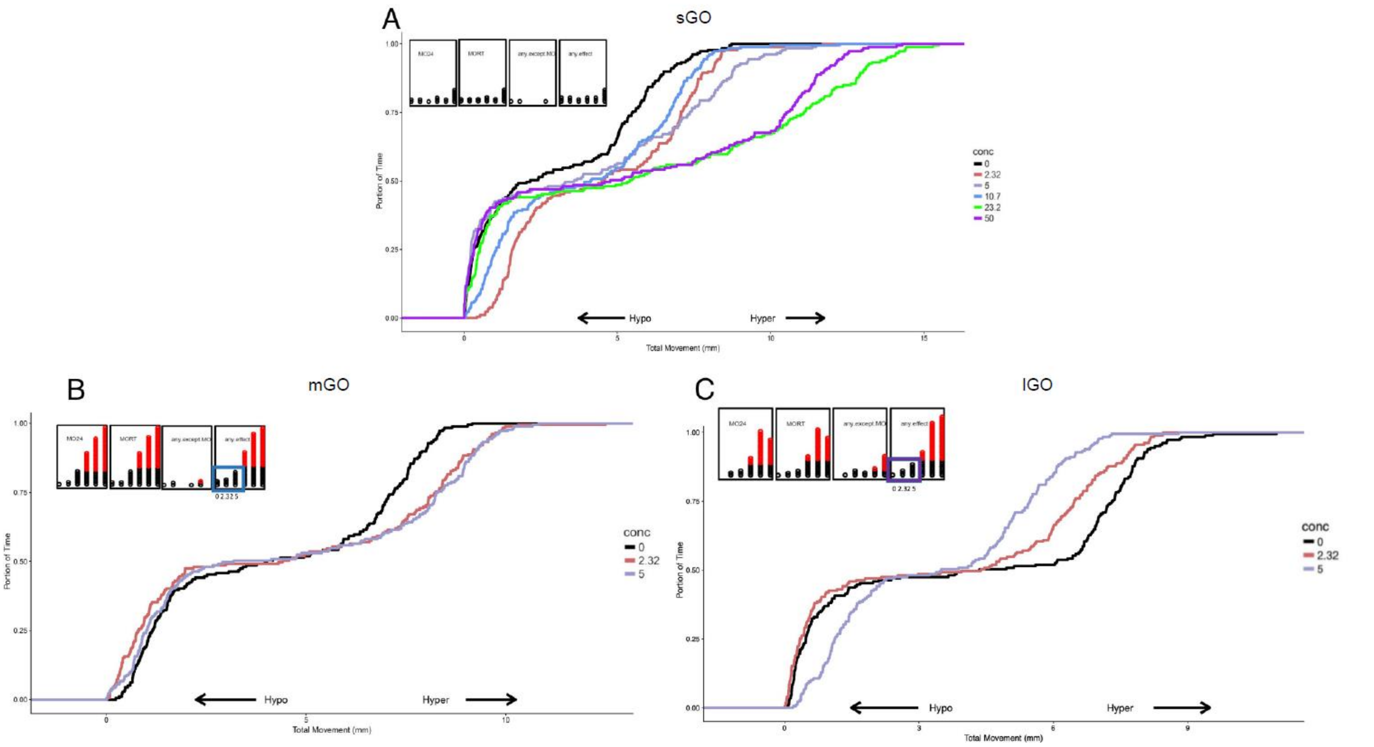

Figure S2. Average movement of 120 hpf zebrafish exposed to GOs. (**A**) sGO. (**B**) mGO. (**C**) lGO.

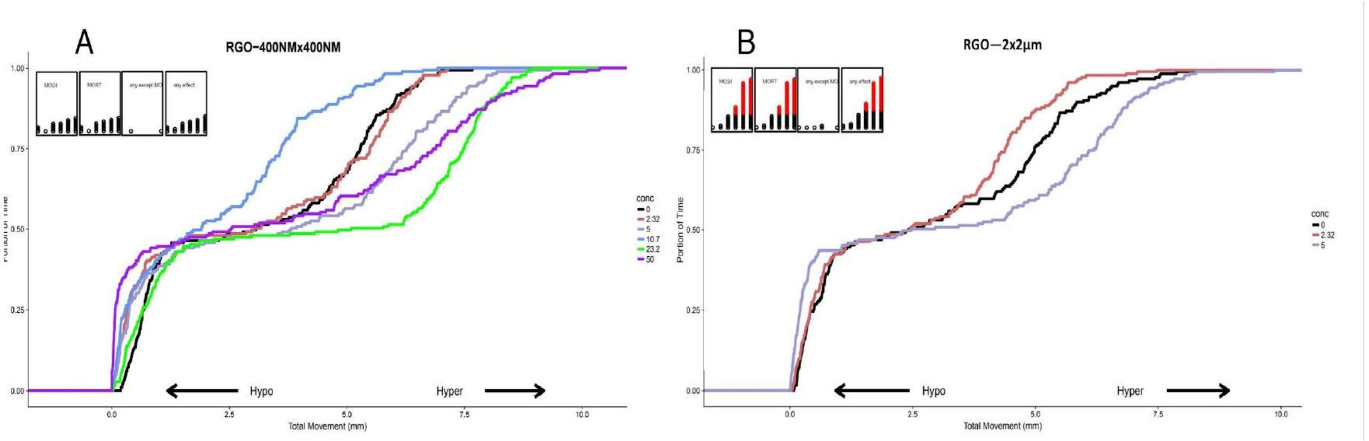

Figure S3. Average movement of 120 hpf zebrafish exposed to rGOs. (**A**) rGO 400 nm × 400 nm. (**B**) rGO 2 μm × 2 μm.

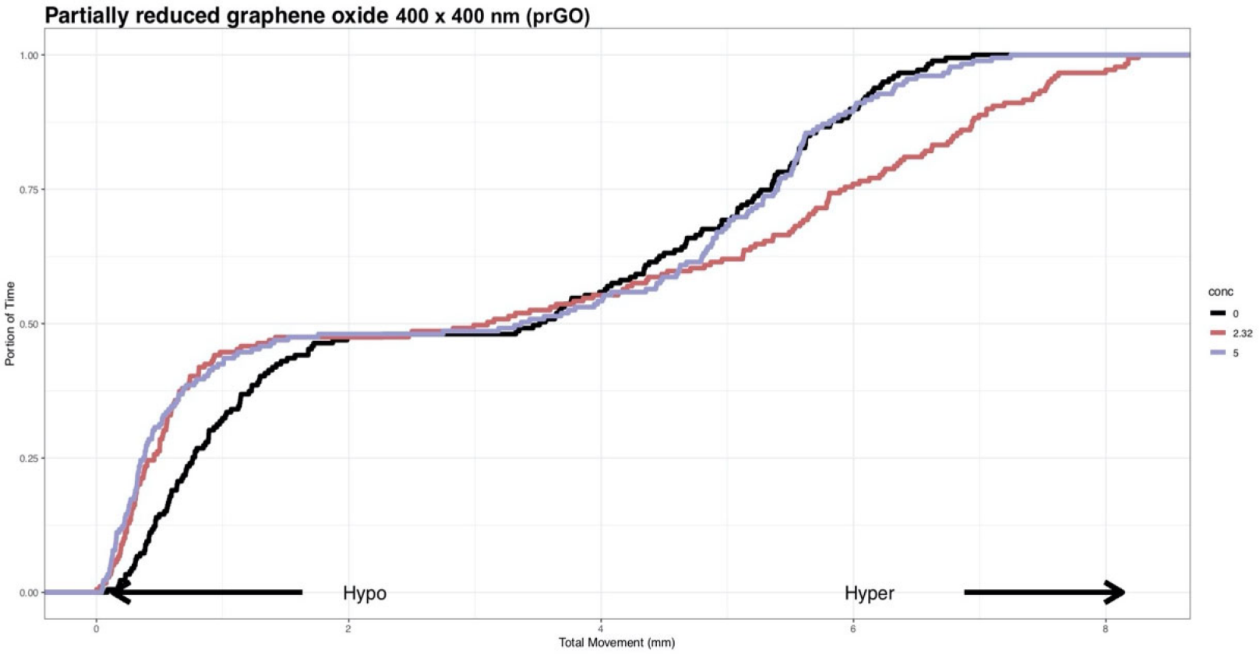

Figure S4. Average movement of 120 hpf zebrafish exposed to prGOs.

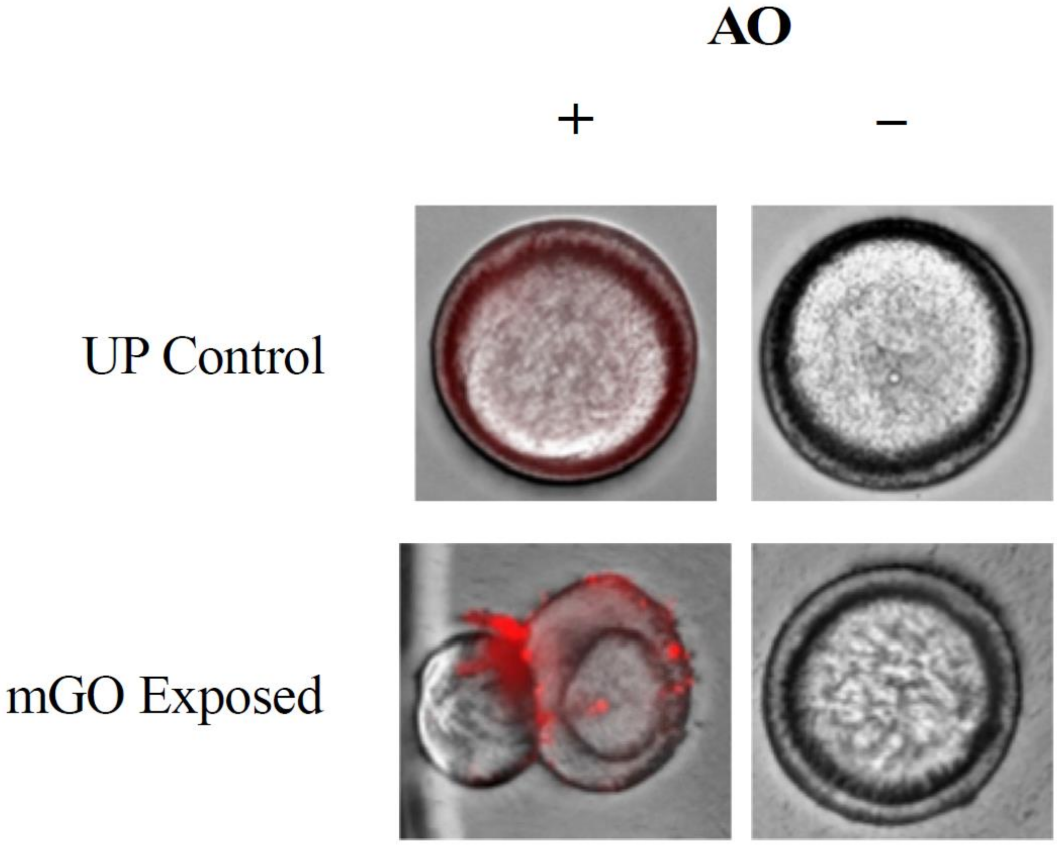

Figure S5. AO in 6 hpf embryos and 24 hpf larvae treated with 50 µg/mL mGO or the control UP water. Top left: Representative UP control embryo incubated at room temperature with AO for 1 h. Widespread signal found in embryo. Top right: Representative embryo developed in UP water. Bottom left: Representative 6 hpf embryo exposed to mGO for 30 min, followed by five washings with UP and AO exposure for 1h. AO accumulation occurred with the lipophilic yolk contents, but there were no localized apoptotic signals detected. Bottom right: Representative embryo exposed to 50 µg/mL mGO for 30 min and washed with UP water five times.

Table S1. Zeta potential of sGO, mGO, lGO dispersed in various media (UltraPure (UP), Embryo Media (EM), or UP with sodium cholate (400 µg /mL).

GO	UP (mV)	EM (mV)	UP + sodium cholate (mV)
sGO	−27.3	−14.7	−27.7
mGO	−38.2	−22.3	−42.6
lGO	−32.0	−18.5	−28.8

Table S2. Average hydrodynamic radius of GOs.

GO	Average hydrodynamic radius (nm)
sGO	314.0
mGO	280.2
lGO	748.7
prGO	246.0
rGO 400 nm × 400 nm	411.7
rGO 2 µm × 2 µm	541.4
